# Landscape of Measurable Residual Disease in Acute Myeloid Leukemia: From Molecular Detection to Clinical Practice

**DOI:** 10.3390/medsci14010123

**Published:** 2026-03-05

**Authors:** Mohammad Shahzaib Qadir, Omer Jamy

**Affiliations:** 1 Medical College, Aga Khan University, Karachi 74200, Pakistan; 2 Division of Hematology/Oncology, Department of Medicine, University of Alabama at Birmingham, Birmingham, AL 35294, USA

**Keywords:** acute myeloid leukemia, measurable residual disease, detection, clinical practice

## Abstract

Measurable residual disease (MRD) has become a central determinant of prognosis and treatment planning in acute myeloid leukemia (AML). MRD assessment is now aided by a wide range of technologies, including next-generation sequencing, PCR-based assays, multiparameter flow cytometry, and emerging approaches such as liquid biopsy platforms and imaging-based detection. These modalities differ in sensitivity, applicability, and interpretive framework, yet each offers distinct advantages in specific disease contexts. Beyond technical issues, MRD is becoming increasingly integrated into clinical practice. In non-intensive treatment settings, where targeted and low-intensity regimens rely on dynamic disease monitoring to guide ongoing management, MRD is increasingly being used to inform therapeutic decisions. In the peri-transplant setting, MRD status influences conditioning strategies, donor selection, and the use of post-transplant interventions. Despite the growing evidence supporting the clinical relevance of MRD across these scenarios, challenges remain regarding standardization, optimal timing of assessment, and the interpretation of discordant results. This review summarizes the full landscape of MRD detection methods and examines the evolving role of MRD in contemporary AML management, emphasizing current applications and areas requiring further refinement.

## 1. Introduction

Acute myeloid leukemia (AML) is a rapidly progressing malignancy that is characterized by the clonal expansion of hematopoietic stem and progenitor cells driven by genetic alterations, resulting in the unchecked accumulation of myeloid blasts that suppress and replace normal hematopoiesis [1]. The prognosis varies widely depending on various patient-related factors, which include comorbidities and age, as well as the underlying molecular and cytogenetic aberrations [2,3,4]. Frequent relapses, which are mostly caused by small populations of chemo-resistant leukemic cells that remain in the bone marrow even after reaching hematologic remission, are typically the cause of poor long-term survival in AML. These residual cells, known as minimal or measurable residual disease (MRD), serve as possible sources of relapse and have generated substantial clinical interest in MRD assessment for AML [5].

As demonstrated in other hematological malignancies, such as chronic myeloid (CML) and acute lymphoblastic leukemia (ALL), MRD evaluation in AML serves multiple clinical purposes. In clinical trials, it can be used as a surrogate endpoint to predict outcomes, help with relapse risk assessment, enable early relapse diagnosis, and evaluate the depth of remission [6,7,8]. Numerous studies have established the prognostic significance of MRD in AML. A comprehensive meta-analysis by Short et al., encompassing over 80 studies, demonstrated that patients who achieved MRD negativity after treatment had a 5-year overall survival rate twice that of MRD-positive patients (68% vs. 34%). This predictive effect held true for all AML subtypes and age groups [9]. Therefore, testing for MRD has emerged as a critical concept in the management of AML [10].

This review provides a comprehensive overview of current MRD detection techniques in AML, highlighting their respective strengths and limitations, while also exploring emerging approaches aimed at addressing existing challenges. We examine the current literature on the clinical relevance of MRD status and its influence on therapeutic decision-making in both intensive and non-intensive treatment settings. Finally, we outline the current applications and persistent limitations of MRD assessment in routine clinical practice.

## 2. Disease Biology of AML and Its Relation to MRD

AML encompasses a diverse group of clonal hematopoietic malignancies driven by recurrent genetic mutations, chromosomal rearrangements, and aberrant signaling pathways [11,12]. Although intensified chemotherapy has improved survival outcomes in AML, relapse remains a significant challenge even after achieving complete remission. Complete remission is defined by the presence of less than 5% blasts in the bone marrow, the absence of circulating blasts and extramedullary disease, an absolute neutrophil count of ≥1.0  ×  10^9^/L, and a platelet count of ≥100  ×  10^9^/L [6].

Standard induction therapy is said to achieve complete remission in approximately 40–70% of patients, but response varies depending on the underlying molecular and cytogenic characteristics [13]. However, the 5-year overall survival is approximately 40% for patients <60 y and this number worsens in the elderly population, with a median 2-year survival of only 20%, indicating that long-term survival is still low despite these remission rates [1]. The fundamental challenge is eradicating leukemic cells without irreparably damaging normal hematopoiesis. This challenge, combined with the rapid proliferative capacity of AML blasts, makes AML one of the most difficult malignancies to treat. The treatment landscape for elderly or unfit AML patients has changed with the introduction of venetoclax (VEN) in combination with hypomethylating agents (HMAs) or low-dose cytarabine (LDAC) as low-intensity regimens. High CR rates have been demonstrated to be induced by VEN plus LDAC or HMAs, and VEN-based regimens are associated with better results compared to LDAC or HMA monotherapy [14,15]. Intensive chemotherapy, often supplemented by targeted agents, can induce cytomorphological remission, but durable disease control remains elusive due to persistence of leukemic clones, especially in intermediate and adverse-risk disease [16].

Although achieving complete remission in AML remains the essential first step towards long-term survival, it does not guarantee a cure, as residual leukemic cells often persist below the threshold of detection and can ultimately drive relapse. These residual populations, if not fully eradicated or adequately controlled by immune surveillance, are capable of reestablishing overt disease [8,17]. While new sophisticated tools have made it possible to discover such cells, conventional morphological evaluation is insufficient to identify them [18]. Consequently, the European LeukemiaNet (ELN) guidelines now recommend MRD testing for all patients who achieve complete remission after intensive chemotherapy to better define relapse risk [6,18]. As a result, MRD is a potent measure of disease status, but its interpretation needs to take clinical context and disease biology into account. Importantly, the MRD threshold compatible with long-term remission may differ across AML subtypes, underscoring the need for integrated risk assessment [19].

## 3. Prognostic Value of MRD

MRD is now slowly being established as an independent prognostic and predictive biomarker in AML [13,20]. Studies have consistently shown the prognostic association between MRD and survival outcomes with an estimated 5-year disease-free survival of 64% in patients who are MRD-negative versus 25% in those who are MRD-positive based on a meta-analysis that was conducted on 48 articles using multiple detection modalities [9]. International efforts have concentrated on standardizing MRD assessment over the years, covering testing frequency, detection techniques, and integration into treatment plans. These efforts culminated in two pivotal ELN consensus documents: the most recent 2025 MRD-focused guidelines, which updated the 2018 and 2021 recommendations [8,21,22], and the 2022 comprehensive AML diagnosis and treatment guidelines, which superseded previous updates from 2010 and 2017 [9,23,24]. These documents now provide the foundation for MRD-guided clinical and research practices.

The particular approach used for MRD detection has a significant impact on analytical sensitivity; the detection limits of various techniques vary by many orders of magnitude. This recognition underlies the transition from the term “minimal residual disease” to “measurable residual disease,” reflecting that the principle test negativity does not imply complete leukemic eradication but rather detection limits of available assays; thus, the field has largely transitioned from the historical term “minimal” to “measurable” [25]. In 2017, the ELN introduced the concept of MRD-negative complete remission, acknowledging that patients testing negative for MRD generally experience superior outcomes compared to MRD-positive patients receiving identical therapy [13,23].

Technological advances, particularly multiparameter flow cytometry (MFC) and molecular assays, have enhanced MRD detection sensitivity and carry a significant predictive value [6,20]. Achieving MRD negativity after consolidation therapy is associated with improved five-year relapse-free survival and overall survival, regardless of the timing of assessment [9]. Conversely, MRD positivity consistently correlates with elevated relapse risk and reduced survival. Early identification of high-risk patients through MRD testing can facilitate pre-emptive interventions, potentially reducing overt hematologic relapse and improving clinical outcomes. Meta-analyses, such as that by Buckley et al. (19 studies, 1431 patients), confirmed that pre-transplant MRD positivity independently predicts reduced disease-free and overall survival, as well as higher relapse risk, regardless of age, conditioning intensity, or detection method [26]. These results highlight how important MRD is in identifying individuals who are at a high risk of adverse outcomes.

Current MRD assessment strategies depend on disease biology. Patients with trackable aberrations, such as acute promyelocytic leukemia, core binding factor (CBF) leukemias, *NPM1* insertions, or BCR-ABL translocations, are typically monitored using quantitative PCR. For almost 60% of patients without such mutations, MFC remains the standard. Large studies, including the NCRI AML17 trial (2450 patients), have validated the prognostic power of MFC for MRD [27]. Nearly 60 suggestions pertaining to MFC MRD, molecular MRD, clinical applications, and future directions, including next-generation sequencing-based MRD (NGS-MRD), were updated in the 2021 ELN MRD guidelines [20,26].

The setting greatly influences how MRD results are interpreted. Genetic subtype, baseline risk, and transplant status all affect prognostic weight. Although accuracy may be harmed by hemodilution or hypocellularity, bone marrow samples are recommended for sensitivity. Emerging data suggest that liquid biopsy approaches, such as cell-free circulating tumor DNA, may provide additional or superior predictive value in select settings [26,27,28,29].

Unresolved questions include how best to integrate MRD across treatment intensities, from high-dose regimens (e.g., 3 + 7, CPX-351) to lower-intensity therapies (e.g., hypomethylating agents, low-dose cytarabine) [13,19]. While targeted interventions such as blinatumomab have proven effective for MRD-positive acute lymphoblastic leukemia [30], similar strategies for AML remain under investigation. Consequently, establishing standardized MRD detection and therapeutic strategies represents a major frontier in AML management.

## 4. Phenotypic and Genetic Methods of Detection of MRD

The identification of MRD in AML utilizes complementary methodologies, each possessing unique strengths and constraints (Table 1). Contemporary detection strategies encompass phenotypic approaches employing MFC-MRD, molecular techniques including quantitative PCR (qPCR-MRD), and emerging high-sensitivity platforms such as digital PCR (dPCR) and next-generation sequencing (NGS) [31,32]. Irrespective of the analytical platform utilized, accurate interpretation necessitates meticulous evaluation of analytical sensitivity and performance characteristics. The limit of detection, representing the minimal signal distinguishable from background interference, and the limit of quantification, denoting the smallest measurable value with acceptable precision, constitute fundamental parameters that must accompany each MRD assessment to establish its clinical relevance [33].

Because of its oligoclonal architecture and underlying biological variability, AML presents unique challenges for MRD evaluation. Strict requirements for specimen quantity and integrity are necessary since different analytical platforms exhibit significant heterogeneity in sensitivity and applicability across various patient groups. For flow cytometric analysis, the established criterion for MRD positivity is ≥10^−3^ CD45^+^ cells [34]. To attain this detection threshold, ELN recommendations specify acquisition of no fewer than 500,000–1,000,000 viable cells from high-quality, undiluted bone marrow specimens processed within 72 h at ambient temperature [20]. Correspondingly, molecular MRD methodologies necessitate sufficient DNA template, with ELN advocating the analysis of ≥10 mL peripheral blood or 5 mL first-draw bone marrow to ensure dependable identification of infrequent leukemic populations [21]. Inadequate specimen quality diminishes analytical sensitivity, thereby elevating the probability of false-negative results. As a result, contextual considerations such as the presence of targetable molecular abnormalities and the disease phenotype influence the choice of MRD methodology. A personalized selection of analytical approaches and assessment timepoints is essential, with MRD increasingly incorporated into therapeutic algorithms to direct treatment intensity and enable precision medicine approaches. Given the complexity and diversity of available MRD detection platforms, a comprehensive understanding of each methodology’s technical principles and clinical applications is essential for optimal implementation in routine practice. The following sections will provide detailed examinations of the primary MRD detection strategies currently employed in AML.

### 4.1. Flow Cytometry

MFC remains the most widely applicable and clinically validated method for MRD detection in AML, particularly for patients without molecular targets suitable for PCR-based monitoring [6,35]. Unlike mutation-specific assays, MFC relies on aberrant antigen-expression patterns of leukemic blasts, making it feasible in more than 90% of AML cases [6,36]. It is widely available across clinical facilities, yields results quickly (24–48 h), and has sensitivities between 10^−3^ and 10^−4^, with refined methods reaching 10^−5^ when there are enough viable cells [37]. The ELN currently defines MRD positivity as ≥0.1% of CD45^+^ leukocytes with a leukemic phenotype, although several studies suggest that lower thresholds may hold prognostic value [38].

MFC-based MRD detection is built upon two complementary analytical strategies: the leukemia-associated immunophenotype (LAIP) approach and the “different-from-normal” (DfN) approach [20]. LAIP tracks patient-specific immunophenotypes defined at diagnosis, allowing longitudinal monitoring of residual leukemic cells, but it is limited by clonal evolution, which alters immunophenotypes in up to 25% of patients [24]. In contrast, DfN identifies abnormal cell populations by comparison with established patterns of normal hematopoiesis, enabling recognition of newly emergent clones but increasing the risk of false positives due to regenerating marrow or clonal hematopoiesis [36]. To address these limitations, the ELN recommends a combined “LAIP-based DfN” strategy, which integrates patient-specific and population-based profiling to improve diagnostic accuracy [6].

ELN recommends antibody panels that include CD34, CD117, CD45, CD33, CD13, CD56, CD7, and HLA-DR for standardized evaluation. When necessary, it also suggests adding markers for monocytic differentiation. To guarantee adequate sensitivity, routine analysis uses CD45-based gating in conjunction with forward and side scatter parameters, acquiring at least 500,000 viable events [23]. Despite progress in harmonization efforts, such as the HARMONEMIA project, inter-laboratory reproducibility remains constrained by variability in antibody panels, cytometer settings, and operator-dependent gating strategies [39].

A major advancement in MFC-MRD has been the immunophenotypic identification of leukemia stem cells (LSCs), typically defined as CD34^+^CD38^−^ populations expressing aberrant markers such as CD123, CD45RA, or CLL-1 [40]. Including LSC-directed panels improves sensitivity, lowers false-negative rates, and exhibits a robust association with the likelihood of recurrence, even in the post-transplant context [41]. Early validation studies demonstrate that LSC-focused MRD assessment significantly outperforms conventional LAIP/DfN-based strategies, with false-negative rates as low as 2–4% [42]. Monitoring the frequency and immunophenotypic evolution of LSCs is emerging as a powerful approach to refine relapse prediction.

A higher relapse rate and lower overall survival rate are clinically predicted by persisting MRD identified by MFC after induction or consolidation therapy. Prognostic importance is also confirmed following allogeneic stem cell transplantation. Large cohort studies show that MRD-positive is linked to negative outcomes even at levels below the traditional 0.1% limit, indicating that thresholds in risk-adapted algorithms need to be re-evaluated [43].

Despite its advantages, several technical and biological challenges limit widespread standardization. MFC requires high-quality, fresh, and undiluted bone marrow samples, as compromised integrity reduces sensitivity and increases false negatives. Peripheral blood monitoring offers a less invasive alternative, but current evidence indicates inferior sensitivity compared to marrow, warranting further prospective validation [38]. Moreover, background signals from regenerating hematopoiesis or clonal hematopoiesis may confound interpretation, necessitating high interpretative expertise. Phenotypic shifts in leukemic clones during therapy further complicate longitudinal surveillance [27].

Looking forward, ongoing efforts to improve MFC-MRD focus on international standardization of antibody panels, semi-quantitative validated assays, and adoption of artificial intelligence-driven analytical platforms to reduce operator subjectivity and enhance reproducibility [44]. Future directions also include broader implementation of LSC-directed surveillance and validation of peripheral blood-based MRD assessment, both of which may increase clinical precision and accessibility.

### 4.2. Polymerase Chain Reaction

Molecular techniques are a cornerstone of MRD assessment in acute myeloid leukemia AML, with quantitative polymerase chain reaction (qPCR) remaining the most widely adopted and standardized method. qPCR can achieve sensitivities in the range of 10^−4^ to 10^−6^, making it one of the most sensitive MRD detection strategies currently available [5,45]. The technique is particularly applicable to AML cases with recurrent, leukemia-specific genomic alterations, including *NPM1* mutations, and CBF leukemias harboring *RUNX1::RUNX1T1* (resulting from t(8;21) rearrangement) or *CBFB::MYH11* fusions (resulting from inv(16) or t(16;16)). It is also used to test for acute promyelocytic leukemia (APL) with *PML::RARA*, and less frequently, *KMT2A::MLLT3*, *DEK::NUP214*, or *BCR::ABL1* rearrangements [6]. Approximately 30–50% of AML patients exhibit such molecular markers, making PCR-based monitoring highly valuable but not universally applicable [46]. In fit patients with *NPM1*-mutated AML, the AML17 trial served as a foundational study to support the prognostic impact of MRD. Compared to their MRD-negative counterparts, patients in MRD-positive complete remission (CR) in this study had a significantly lower 3-year survival rate (24% vs. 75%) and a significantly higher risk of relapse (82% vs. 30%) [47].

In an AMLSG study, patients with CBF-AML who had less than a 3-log reduction in MRD from bone marrow after two treatment cycles showed a 2-year CIR of 51% versus 28% using the same MRD thresholds. MRD was not prognostic for overall survival (OS) in this study [48]. However, a study from MD Anderson Cancer Center reported that patients with CBF-AML who achieved PCR levels below 0.1% in bone marrow after induction had significantly superior OS compared with those with higher MRD values (5-year OS 85% versus 61%; *p*  =  0.01). The differing results among studies may be related to variations in treatment intensity, as the MD Anderson study used a more intensive induction regimen (either FLAG-Ida or FLAG-GO) [49].

Reverse transcription qPCR (RT-qPCR) is particularly effective for fusion transcripts and *NPM1* mutations due to their high expression levels. In this context, RNA is preferred over DNA, as fusion breakpoints often occur in intronic regions, whereas transcript detection ensures higher specificity and sensitivity [50]. Standardized assays for *RUNX1::RUNX1T1* and *CBFB::MYH11* fusion transcripts, as well as *NPM1* type A, B, and D insertions, are well established and allow reliable MRD quantification. Persistent detection of these markers after chemotherapy strongly correlates with an increased risk of relapse [51,52].

Despite its broad utility, qPCR has notable limitations. First, it only provides relative quantification based on threshold cycle (Ct) values normalized to control genes such as *ABL1* [53]. Differences in control genes, or laboratory practices can lead to variability, and interlaboratory studies have documented discordant results, particularly in *NPM1* testing where false positives can arise during reverse transcription. This underscores the need for ongoing assay standardization and external quality control initiatives [54].

qPCR can also be applied to other recurrent mutations, including *FLT3*-ITD, *IDH1/2*, and overexpression of *WT1* or *EVI1*. However, these markers pose additional challenges. In the recent Phase III MORPHO study, 356 patients were tested for *FLT3*-ITD using a PCR-based NGS technique; 700 ng of genomic DNA was amplified by 25 cycles of PCR using primers flanking exons 14 and 15 of *FLT3*, and the amplicons were analyzed by NGS. The lower limit of detection matched an *FLT3*-ITD variant allele frequency of 5 × 10^−5^. Any *FLT3*-ITD signal exceeding this threshold, defined as at least three variant reads and measurable down to 1 × 10^−6^, was classified as detectable MRD regardless of whether it matched the diagnostic mutation [55]. Internal tandem duplication mutations of FLT3 (*FLT3*-ITD) confer an increased relapse risk and are unstable across disease evolution, exhibiting patient-specific variability in insertion size and location, and thus require individualized assays with limited reproducibility [56]. Concurrently, *WT1* is overexpressed in the majority of AML cases, but its physiological expression in normal hematopoietic tissue generates background noise, resulting in poor sensitivity and specificity [57]. For these reasons, ELN guidelines recommend using such markers only when no other molecular or cytometric targets are available [20].

Digital droplet PCR (ddPCR) has emerged as a complementary technology with the potential to overcome some of these limitations. Unlike qPCR, ddPCR enables absolute quantification without reliance on standard curves, improving precision and reproducibility [58]. It has demonstrated utility in detecting rare *NPM1* variants beyond the canonical A, B, and D subtypes, and has also been explored for *IDH1/2* mutations and other AML-associated genetic lesions [59]. Novel approaches, such as double drop-off ddPCR (DDO-ddPCR), further enhance sensitivity and allow the analysis of both bone marrow and peripheral blood, as well as cell-free DNA (cfDNA), offering a less invasive avenue for MRD monitoring [60]. Nevertheless, ddPCR still requires custom assay development, limiting its widespread standardization at present.

Overall, PCR-based MRD monitoring represents a sensitive and clinically validated approach in AML, particularly when applied to well-characterized molecular lesions such as *NPM1* mutations and CBF-associated fusions. Its prognostic value is firmly established, but challenges including marker applicability, assay variability, and the need for technical expertise highlight the importance of ongoing international standardization efforts and the integration of complementary technologies such as MFC and next-generation sequencing (NGS).

### 4.3. Next-Generation Sequencing

NGS has emerged as a promising tool for MRD monitoring in AML, with the key advantage of broad applicability across diverse genomic alterations [32]. Unlike PCR-based methods that are limited to predefined targets, NGS can theoretically detect any somatic mutation, enabling the simultaneous analysis of multiple mutations in a single assay [61]. Different platforms are available, including whole-genome sequencing (WGS), whole-exome sequencing (WES), and targeted sequencing panels, with the latter being most commonly applied in MRD testing due to its balance of sensitivity, cost, and clinical relevance [62].

NGS-based MRD analysis quantifies residual leukemic clones through VAF estimation. Mutations with similar VAFs are inferred to belong to the same clone, while higher-frequency variants typically reflect founder clones. Tracking VAF dynamics provides insight into clonal evolution, treatment response, and resistance mechanisms. Decreasing VAFs after therapy suggest effective cytoreduction, whereas rising VAFs may indicate clonal expansion and impending relapse [62].

Despite its conceptual advantages, conventional NGS platforms are limited by intrinsic error rates from PCR amplification and sequencing, typically ranging between 0.1% and 1%. Consequently, most assays have reliable sensitivity only down to ~1–2% VAF, which is insufficient for detecting very low-level MRD [61]. Insertions and deletions (e.g., *NPM1* or *FLT3*-ITD) can sometimes be detected with higher sensitivity than single-nucleotide variants (SNVs), but the general detection threshold remains higher than that of qPCR or digital PCR [44,63]. Efforts to improve sensitivity include the incorporation of unique molecular identifiers (UMIs), duplex sequencing, and other error-corrected sequencing strategies. These approaches can reduce false positives, enhance reproducibility, and, in some studies, achieve sensitivities approaching 10^−6^. However, the increased computational burden, cost, and lack of standardized protocols currently limit their widespread clinical adoption [64,65].

From a biological perspective, the interpretation of NGS results poses additional challenges. Many mutations detected in complete remission represent age-related clonal hematopoiesis (CH) rather than persistent leukemia. Mutations in *DNMT3A*, *TET2*, and *ASXL1* (collectively referred to as DTA mutations) are particularly common in CH and are generally excluded from MRD analyses, as their persistence does not correlate with relapse risk [6]. Similarly, mutations in *SRSF2*, *IDH1*, and *IDH2* may also reflect CH and not active leukemia. In contrast, persistence of non-DTA mutations, even at low VAF (<2.5%), has been consistently associated with increased relapse risk and inferior survival [66].

Several studies have highlighted the prognostic utility of NGS-based MRD detection. For example, detection of *FLT3*-ITD mutations by NGS at very low VAF thresholds has been shown to predict post-transplant relapse and inferior survival outcomes [67]. These findings support the use of NGS as a complementary tool to established techniques such as qPCR and multiparameter flow cytometry (MFC), particularly in patients without canonical molecular MRD markers. Patients who had detectable *NPM1* MRD in their blood prior to transplant using NGS had a 3-year OS of 35% in a Pre-Measure study, compared to 66% in those who tested negative [63].

Nonetheless, major hurdles remain. The relatively low sensitivity of routine NGS compared to qPCR means that some high-risk patients may escape detection. The lack of robust head-to-head comparisons between NGS and qPCR for key markers such as *NPM1* complicates clinical implementation, as most evidence-based treatment decisions are grounded in qPCR data [44].

To guide clinical decision-making, a hierarchical monitoring algorithm can be used based on the baseline molecular profile of the patient, aligned with the new 2025 ELN-DAVID consensus recommendations [22]. For patients harboring *NPM1* mutations or CBF fusions (*RUNX1::RUNX1T1, CBFB::MYH11*), high-sensitivity RT-qPCR remains the ideal standard for surveillance due to its superior sensitivity (10^−4^ to 10^−6^) and standardization. In the absence of these core markers, patients with other recurrent mutations, such as *FLT3*-ITD, should be monitored using validated NGS or specific PCR assays, often in conjunction with MFC to account for clonal instability or variable sensitivity. Finally, for patients lacking trackable molecular markers, MFC serves as the primary monitoring modality, utilizing LAIP or DfN approaches. This stratified strategy ensures that the most sensitive and specific tool is utilized for each biological subgroup [13,20,22].

## 5. Novel MRD Detection Methods

### 5.1. Liquid Biopsy

One promising minimally invasive method for detecting MRD in AML is liquid biopsy [68]. This method has the potential to replace bone marrow aspirations by examining blood-derived components like circulating tumor cells (CTCs), circulating RNAs (cRNAs), and circulating tumor DNA (ctDNA) [69]. Its prognostic significance is highlighted by the significant correlation found between the detection of CTCs in AML and relapse-free survival. CtDNA is the most researched liquid biopsy analyte, especially in solid tumors where it is a trustworthy biomarker [70]. The term “ctDNA” refers to the fragmented cell-free DNA (cfDNA) that cancerous cells release into the bloodstream. Although apoptosis causes cfDNA to be present in healthy people as well, cancer patients have significantly higher levels of cfDNA due to increased cellular turnover. In AML, elevated cfDNA concentrations have been associated with MRD positivity and higher relapse risk, and ctDNA analysis can be performed using NGS [71]. Despite its promise, several limitations remain. Sample processing must occur rapidly to prevent white blood cell lysis, complicating logistics. In addition, ctDNA-based MRD assessment may yield false negatives by missing leukemic subclones with low-variant allele frequencies or false positives due to clonal hematopoiesis-associated mutations [72].

### 5.2. CT-Scan/PET CT

Noninvasive imaging modalities, particularly positron emission tomography (PET/CT), have also been explored as alternatives for prognostic indicators of disease activity. ^18F^FDG PET, which measures glucose metabolism, has shown some potential for detecting leukemic infiltration, but its specificity is limited [73].

More promising is the use of 3′-deoxy-3′-^18F^fluorothymidine (FLT) PET, which reflects cellular proliferation through an uptake dependent on thymidine kinase 1 (TK-1) activity. TK-1 is markedly overexpressed in leukemic blasts, leading to enhanced FLT accumulation. Pilot studies demonstrated that AML patients exhibit significantly higher FLT uptake in bone marrow and the spleen compared with controls, and interim FLT PET after induction therapy distinguished responders from patients with residual disease. In particular, low bone marrow uptake correlated with CR, while persistently elevated uptake predicted refractory disease and early relapse [74,75]. An important advantage of FLT PET is its ability to provide whole-body evaluation of the hematopoietic compartment, thereby overcoming the limitations of site-restricted bone marrow biopsies.

A prospective multicenter study further evaluated the predictive value of FLT PET/CT after induction chemotherapy [76]. Although the overall predictive accuracy for CR or residual disease was comparable to nadir bone marrow biopsy, FLT PET offered additional insights into marrow heterogeneity that were not captured by single-site biopsies. Despite its promise, FLT PET/CT faces several challenges. Normal regenerating hematopoietic cells also demonstrate FLT uptake, complicating discrimination from leukemic proliferation. Small sample sizes, a lack of standardized imaging protocols, and absence of universally accepted interpretation criteria further limit its clinical application [75]. Nonetheless, preliminary findings indicate that interim FLT PET/CT may enhance early prediction of treatment response and relapse risk. Further studies are required to assess the role of PET/CT imaging in detecting residual disease.

## 6. Role of MRD in Detection of Non-Intensive Treatments

The frequency of MRD monitoring varies by practice; the ELN consensus advises MRD monitoring at a minimum of three points: at diagnosis, following two cycles of standard induction or consolidation chemotherapy, and upon completion of treatment. This recommendation keeps in mind the evolution of molecular MRD markers and the improving sensitivity of MRD-detection techniques [23]. In the first 24 months following treatment completion, follow-up testing is also advised for well-characterized AML phenotypes (such as *NPM1*-mutant, CBF, and APL) by bone marrow sampling every three months or peripheral blood every four to six weeks [20]. For patients receiving low-intensity therapy, the tracking of MRD, MFC, and qPCR of previously identified molecular markers (such as *NPM1*) in bone marrow every three treatment cycles until MRD response, and then every one to three months in the peripheral blood, is advised [77].

The introduction of venetoclax (VEN) in combination with hypomethylating agents (HMA) or low-dose cytarabine (LDAC) as low-intensity regimens has transformed the therapeutic landscape for elderly or unfit AML patients. Therefore, the role of the MRD status in these populations has become of growing interest [14,78,79]. The VIALE-A trial established that VEN combined with azacitidine (AZA) significantly improved CR and MRD-negative rates compared with AZA alone, with up to 41% of patients achieving MRD negativity by MFC and particularly high rates in *NPM1*-mutated cases. Sequential cycles deepened the MRD response, with nearly half of patients achieving negativity within four cycles [77,80].

Several studies have confirmed that MRD negativity following VEN-based regimens correlates with superior outcomes. VEN plus AZA or LDAC have been shown to induce high CR rates with meaningful proportions of patients achieving MRD clearance. Long-term follow-up revealed durable remissions and favorable survival in MRD-negative patients [15]. Similarly, a decitabine–VEN regimen achieved MRD negativity in over half of evaluable patients within two months, which translated into prolonged relapse-free, event-free, and overall survival. Importantly, early MRD clearance (by two months) was associated with the most pronounced survival benefit. MRD negativity after the first or second cycle of decitabine–VEN predicted longer survival [81].

A study identified a threshold of 0.1% by MFC as most predictive for relapse-free and overall survival [82]. In patients with *NPM1*-mutated AML treated with VEN-HMA, Othman et al. showed stepwise increases in molecular MRD clearance across treatment cycles, which strongly correlated with improved outcomes [78]. Additional studies have demonstrated that VEN-based regimens yield high rates of MRD negativity and that achieving this milestone is consistently associated with superior survival compared with MRD-positive remissions [77].

MRD monitoring has also been investigated with hypomethylating agent maintenance in patients with CBF-AML. In this setting, reductions in MRD, particularly when achieved early and at low levels, predicted better clinical outcomes. These findings collectively suggest that even with non-intensive therapy, MRD status retains independent prognostic significance irrespective of treatment intensity [83].

The ELN–DAVID AML MRD Working Group has proposed that MRD monitoring be incorporated into the management of patients receiving low-intensity regimens. Recommended time points for MRD assessment include bone marrow evaluation after the first, second, fourth, and seventh cycles [84]. However, while MRD negativity clearly identifies a subgroup with improved outcomes, its role in guiding treatment discontinuation or de-escalation remains uncertain, as some studies did not demonstrate a survival benefit when treatment cessation was based on MRD results. Evidence strongly supports MRD as a prognostic marker in patients receiving VEN-based or other low-intensity regimens. Ongoing prospective trials are needed to refine its role in clinical decision-making, including the potential for therapy adaptation based on MRD kinetics.

## 7. Role of MRD in Peri-Transplant Setting

The prognostic relevance of MRD in the context of allogeneic hematopoietic stem cell transplantation (allo-HSCT) has been firmly established [85]. Patients transplanted in MRD-negative CR consistently demonstrate superior survival and lower relapse rates compared with those who are MRD-positive at the time of transplant [86,87]. Retrospective analyses, including large cohorts, show a graded survival difference across three categories, MRD-negative CR, MRD-positive CR, and active disease at transplant, with outcomes progressively worsening in this sequence. Importantly, these findings hold true both in first and second remission (CR1 and CR2), although long-term survival is occasionally observed even in patients undergoing transplantation with persistent MRD [88,89].

The utility of MRD to refine transplant decision-making is particularly relevant in intermediate-risk AML, where the optimal consolidative strategy remains controversial. Prospective and retrospective studies, such as the GIMEMA AML1310, demonstrate that patients with persistent MRD derive significant benefit from allo-HSCT, while MRD-negative patients may achieve comparable outcomes with chemotherapy or autologous HSCT [90]. In *NPM1*-mutated AML, insufficient molecular clearance (<4-log reduction) predicts benefit from allo-HSCT, whereas patients achieving deeper reductions show limited additional advantages [27,91]. Similarly, in core-binding factor AML, transplantation appears beneficial only for patients failing to reach defined molecular thresholds (e.g., <3-log reduction in *RUNX1::RUNX1T1* transcripts), whereas those achieving deeper responses maintain excellent survival without transplant [92].

In contrast, for high-risk genetic subgroups such as FLT3-ITD-mutated AML (without co-occurring *NPM1* mutation), allogeneic transplantation remains standard irrespective of MRD status, although the role of high-sensitivity *FLT3*-MRD assays in decision-making is under investigation [6]. The recent phase III MORPHO trial, which used a PCR-based NGS technique to test for *FLT3*-ITD, demonstrated the critical impact of assay thresholds. MRD in Pre-HCT patients at or above the stratification threshold of 1 × 10^−4^ was present in 75 of 356 patients (21.1%), increasing the sensitivity to 1 × 10^−6^, which more than doubled the detection rate to 164 of 356 patients (46.1%). Post-HCT, MRD detected at greater than 1 × 10^−6^ persisted in 71 of 356 patients (19.9%), including 16 individuals (4.5%) who only became MRD-positive post HCT. Overall, 180 of 356 patients (50.6%) exhibited measurable *FLT3*-MRD during the peri-transplant period, underscoring the frequency of residual molecular disease [55]. Emerging evidence also suggests that MRD positivity in favorable-risk AML may identify a subset who could benefit from allo-HSCT, but prospective randomized studies are needed to clarify this question.

MRD status also informs the choice of conditioning regimen. Multiple studies report that patients with detectable MRD prior to allo-HSCT experience high relapse rates regardless of conditioning intensity; however, several large cohorts suggest that myeloablative conditioning (MAC) may mitigate relapse risk more effectively than reduced-intensity conditioning (RIC), particularly in younger patients [93,94]. Conversely, in MRD-negative patients, conditioning intensity appears to have limited impact on survival, raising the possibility that toxicity from MAC could be avoided in this group [95]. These data support the ELN recommendation to favor MAC in MRD-positive patients whenever feasible [20]. Pretransplant MRD may also influence donor selection. Comparative analyses indicate that MRD-positive patients undergoing haploidentical HSCT (haplo-HSCT) may experience lower relapse rates and superior survival than those receiving HLA-matched sibling donor transplants, likely due to a stronger graft-versus-leukemia effect [96].

Post-transplant MRD monitoring provides a dynamic means of risk assessment and is central to early relapse detection and risk-adapted intervention, with its clinical utility closely dependent on the sensitivity and applicability of the detection modality [20,22]. Molecular MRD assessment using RT-qPCR or ddPCR remains the most sensitive and well-validated approach for post-transplant surveillance in patients with trackable lesions such as *NPM1*, CBF rearrangements, and *FLT3*-ITD. The rising transcript levels or failure to achieve molecular clearance frequently preceded overt relapse and enabled pre-emptive intervention [97]. In patients lacking molecular markers, serial MFC provides broad applicability for post-transplant monitoring and is recommended during the first year of follow-up after alloHCT according to the updated 2025 ELN guidelines [22]. However, its use in the post-transplant context has been limited by lower analytical sensitivity compared with PCR-based assays, due to challenges related to immunophenotypic shifts and uncertainty regarding interpretation in the setting of mixed donor–host chimerism evolution [98]. While qPCR for specific targets remains the most appropriate method due to sensitivity, novel technologies are emerging to address the limitations of invasive bone marrow sampling in the post-transplant setting. For instance, studies utilizing microfluidic devices coated with antibodies against leukemic (CD33, CD34, CD117) and aberrant (CD7, CD56) antigens have demonstrated high specificity (88–99%) for circulating leukemic cells. This microfluidic approach has shown potential to detect signs of relapse more rapidly than conventional bone marrow-based assays [99].

Persistent or recurrent MRD detected by any modality following transplantation is associated with high relapse risk, and several MRD-directed strategies are under evaluation. Hypomethylating agents, such as azacitidine or decitabine, have demonstrated efficacy as pre-emptive therapies to delay or prevent hematologic relapses in patients with detectable MRD post-HSCT [100]. Targeted maintenance approaches have also gained traction, particularly in *FLT3*-ITD-mutated AML, where agents such as sorafenib, gilteritinib (MRD+), and quizartinib have found to improve patient outcomes as maintenance therapy after allo-HSCT [55,101,102]. Donor lymphocyte infusions (DLI), often combined with reduction in immunosuppression, remain an established intervention for MRD relapse, with pre-emptive administration increasingly favored over salvage approaches [103].

Collectively, MRD serves as a critical prognostic marker in the peri-transplant setting and increasingly informs decisions regarding the indication for allo-HSCT, donor selection, and post-transplant maintenance. While robust data demonstrates its value in intermediate-risk and molecularly defined AML subsets, ongoing prospective trials are required to refine MRD-driven transplant strategies and fully integrate them into clinical practice.

## 8. Conclusions

MRD has become a critical tool for refining prognosis and guiding management in AML. Advances across multiple detection platforms, including MFC, PCR, and NGS, have expanded the ability to quantify residual disease with increasing precision. Newer approaches, such as liquid biopsy and imaging-based assessment, may further broaden MRD evaluation, although their roles require additional validation through future studies. Despite this progress, no single technique is universally applicable, and variation in sensitivity, standardization, and interpretation continues to limit uniform adoption. Integrating results across complementary modalities remains essential, particularly in heterogeneous disease contexts.

The clinical impact of MRD is now evident across diverse treatment settings. In non-intensive regimens, MRD dynamics help characterize depth of response and may support individualized treatment strategies. In the peri-transplant setting, MRD status increasingly influences transplant decisions, conditioning intensity, and the use of post-transplant interventions. However, the application of MRD to direct therapy remains inconsistent, and evidence from prospective trials is needed to define when MRD-guided strategies improve outcomes. Continued efforts to standardize testing and evaluate MRD-directed interventions will be central to incorporating MRD into routine AML care. As detection methods evolve and therapeutic options expand, MRD has the potential to support more precise risk stratification and more adaptive treatment approaches across the AML spectrum.

## Figures and Tables

**Table 1:** Strengths and limitations of current MRD detection methods for patients with AML.

Method	Sensitivity (Approx.)	Case Applicability	Advantages	Disadvantages
**Multiparameter Flow Cytometry (MFC; LAIP/DfN)**	10^−3^–10^−5^	>90%	Rapid turnaround (~24 h); Broad applicability; Relatively inexpensive; Widely available; Allows morphological correlation.	Requires fresh samples; Interpretation requires expertise; Limited inter-laboratory standardization; Immunophenotypic shifts may cause false negatives.
**RT-qPCR/ddPCR**	10^−4^–10^−6^	40–50% (restricted to known molecular targets)	High analytical sensitivity and specificity; Well standardized; Quantitative (especially ddPCR); Widely available.	Applicable only to known mutations/fusions; Time-consuming; Single-lesion assays; Misses variants outside primer regions.
**Next-Generation Sequencing (NGS)**	10^−2^–10^−6^ (platform-dependent)	Nearly all patients	Comprehensive mutational profiling; Detects multiple mutations simultaneously; Captures clonal evolution; High multiplexing potential.	Expensive and time-consuming; Not yet standardized; Lower sensitivity on standard platforms; May misclassify CHIP mutations as residual disease; Requires advanced bioinformatics.
**Liquid Biopsy (ctDNA, CTCs, cfDNA)**	10^−3^–10^−5^ (depending on analyte and platform)	Potentially all patients	Minimally invasive (blood-based); Enables serial monitoring; Reflects total body disease burden; Detects molecular relapse before hematologic recurrence; Integrates with NGS or PCR-based assays.	Requires rapid processing to prevent leukocyte lysis; Possible false negatives (low VAF subclones); Possible false positives due to CHIP-associated mutations; Limited standardization and validation in AML.
**PET/CT (especially FLT PET)**	Semi-quantitative (functional imaging; not molecular)	Broad (whole-body evaluation)	Noninvasive, whole-body assessment; Detects extramedullary and marrow disease; FLT PET correlates with proliferative activity; Differentiates responders from refractory cases; Provides spatial insight into marrow heterogeneity.	Limited specificity (uptake in regenerating marrow); Expensive and not widely available; Lack of standardized imaging protocols; Interpretation criteria not universally established; Currently adjunctive rather than standalone MRD tool.

## Data Availability

No new data were created or analyzed in this study. Data sharing is not applicable to this article.

## Abbreviations

AML	Acute Myeloid Leukemia
MRD	Measurable Residual Disease
MFC	Multiparameter Flow Cytometry
LAIP	Leukemia-Associated Immunophenotype
DfN	Difference from Normal
RT-qPCR	Reverse Transcription Quantitative Polymerase Chain Reaction
ddPCR	Droplet Digital Polymerase Chain Reaction
NGS	Next-Generation Sequencing
CHIP	Clonal Hematopoiesis of Indeterminate Potential
ctDNA	Circulating Tumor DNA
cfDNA	Cell-Free DNA
CTCs	Circulating Tumor Cells
VAF	Variant Allele Frequency
PET/CT	Positron Emission Tomography/Computed Tomography
FLT	3′-Deoxy-3′-^18^F-Fluorothymidine

## References

[1] Aitken M.J.L., Ravandi F., Patel K.P., Short N.J. (2021). Prognostic and therapeutic implications of measurable residual disease in acute myeloid leukemia. J. Hematol. Oncol..

[2] De Kouchkovsky I., Abdul-Hay M. (2016). Acute myeloid leukemia: A comprehensive review and 2016 update. Blood Cancer J..

[3] Arber D.A., Orazi A., Hasserjian R.P., Borowitz M.J., Calvo K.R., Kvasnicka H.M., Wang S.A., Bagg A., Barbui T., Branford S. (2022). International Consensus Classification of Myeloid Neoplasms and Acute Leukemias: Integrating morphologic, clinical, and genomic data. Blood.

[4] Arber D.A., Hasserjian R.P., Orazi A., Mathews V., Roberts A.W., Schiffer C.A., Roug A.S., Cazzola M., Döhner H., Tefferi A. (2022). Classification of myeloid neoplasms/acute leukemia: Global perspectives and the international consensus classification approach. Am. J. Hematol..

[5] Vonk C.M., Al Hinai A.S.A., Hanekamp D., Valk P.J.M. (2021). Molecular minimal residual disease detection in acute myeloid leukemia. Cancers.

[6] Döhner H., Wei A.H., Appelbaum F.R., Craddock C., DiNardo C.D., Dombret H., Ebert B.L., Fenaux P., Godley L.A., Hasserjian R.P. (2022). Diagnosis and management of AML in adults: 2022 recommendations from an international expert panel on behalf of the ELN. Blood.

[7] Dekker S.E., Rea D., Cayuela J.M., Arnhardt I., Leonard J., Heuser M. (2023). Using measurable residual disease to optimize management of AML, ALL, and chronic myeloid leukemia. Am. Soc. Clin. Oncol. Educ. Book.

[8] Short N.J., Zhou S., Fu C., Berry D.A., Walter R.B., Freeman S.D., Hourigan C.S., Huang X., Nogueras-Gonzalez G., Hwang H. (2020). Association of Measurable Residual Disease with Survival Outcomes in Patients with Acute Myeloid Leukemia: A Systematic Review and Meta-analysis. JAMA Oncol..

[9] Short N.J., Fu C., Berry D.A., Walter R.B., Freeman S.D., Hourigan C.S., Huang X., Nogueras-Gonzalez G., Hwang H., Qi X. (2022). Association of hematologic response and assay sensitivity on the prognostic impact of measurable residual disease in acute myeloid leukemia: A systematic review and meta-analysis. Leukemia.

[10] El Chaer F., Hourigan C.S., Zeidan A.M. (2023). How I treat AML incorporating the updated classifications and guidelines. Blood.

[11] Vosberg S., Greif P.A. (2019). Clonal evolution of acute myeloid leukemia from diagnosis to relapse. Genes. Chromosomes Cancer.

[12] Short N.J., Rytting M.E., Cortes J.E. (2018). Acute myeloid leukaemia. Lancet.

[13] Döhner H., Estey E., Grimwade D., Amadori S., Appelbaum F.R., Büchner T., Dombret H., Ebert B.L., Fenaux P., Larson R.A. (2017). Diagnosis and management of AML in adults: 2017 ELN recommendations from an international expert panel. Blood.

[14] Wei A.H., Montesinos P., Ivanov V., DiNardo C.D., Novak J., Laribi K., Kim I., Stevens D.A., Fiedler W., Pagoni M. (2020). Venetoclax plus LDAC for newly diagnosed AML ineligible for intensive chemotherapy: A phase 3 randomized placebo-controlled trial. Blood.

[15] DiNardo C.D., Pratz K., Pullarkat V., Jonas B.A., Arellano M., Becker P.S., Frankfurt O., Konopleva M., Wei A.H., Kantarjian H.M. (2019). Venetoclax combined with decitabine or azacitidine in treatment-naive, elderly patients with acute myeloid leukemia. Blood.

[16] Döhner H., Wei A.H., Löwenberg B. (2021). Towards precision medicine for AML. Nat. Rev. Clin. Oncol..

[17] Hart J., Shirakawa S., Trujillo J., Res E.F.I. (1969). The mechanism of induction of complete remission in acute myeloblastic leukemia in man. Cancer Res..

[18] Cloos J., Ngai L.L., Heuser M. (2023). Understanding differential technologies for detection of MRD and how to incorporate into clinical practice. Hematology.

[19] Walter R.B., Ofran Y., Wierzbowska A., Ravandi F., Hourigan C.S., Ngai L.L., Venditti A., Buccisano F., Ossenkoppele G.J., Roboz G.J. (2021). Measurable residual disease as a biomarker in acute myeloid leukemia: Theoretical and practical considerations. Leukemia.

[20] Heuser M., Freeman S.D., Ossenkoppele G.J., Buccisano F., Hourigan C.S., Ngai L.L., Tettero J.M., Bachas C., Baer C., Béné M.-C. (2021). 2021 Update on MRD in acute myeloid leukemia: A consensus document from the European LeukemiaNet MRD Working Party. Blood.

[21] Walter W., Pfarr N., Meggendorfer M., Jost P., Haferlach T., Weichert W. (2022). Next-generation diagnostics for precision oncology: Preanalytical considerations, technical challenges, and available technologies. Semin. Cancer Biol..

[22] Cloos J., Valk P.J.M., Thiede C., Döhner K., Roboz G.J., Wood B.L., Walter R.B., Wang S.A., Wierzbowska A., Wei A.H. (2025). 2025 Update on MRD in Acute Myeloid Leukemia: A Consensus Document from the ELN-DAVID MRD Working Party. Blood.

[23] Schuurhuis G.J., Heuser M., Freeman S., Béné M.-C., Buccisano F., Cloos J., Grimwade D., Haferlach T., Hills R.K., Hourigan C.S. (2018). Minimal/measurable residual disease in AML: A consensus document from the European LeukemiaNet MRD Working Party. Blood.

[24] Sui J.N., Chen Q.S., Zhang Y.X., Sheng Y., Wu J., Li J.M., Weng X.Q., Chen B. (2019). Identifying leukemia-associated immunophenotype-based individualized minimal residual disease in acute myeloid leukemia and its prognostic significance. Am. J. Hematol..

[25] Goldman J.M., Gale R.P. (2014). What does MRD in leukemia really mean?. Leukemia.

[26] Buckley S.A., Wood B.L., Othus M., Hourigan C.S., Ustun C., Linden M.A., DeFor T.E., Malagola M., Anthias C., Valkova V. (2017). Minimal residual disease prior to allogeneic hematopoietic cell transplantation in acute myeloid leukemia: A meta-analysis. Haematologica.

[27] Freeman S.D., Hills R.K., Virgo P., Khan N., Couzens S., Dillon R., Gilkes A., Upton L., Nielsen O.J., Cavenagh J.D. (2018). Measurable residual disease at induction redefines partial response in acute myeloid leukemia and stratifies outcomes in patients at standard risk without NPM1 mutations. J. Clin. Oncol..

[28] Rodríguez-Arbolí E., Orvain C., Othus M., Walter R.B. (2022). Significance of measurable residual disease in adults with secondary acute myeloid leukemia undergoing allogeneic hematopoietic cell transplantation. Bone Marrow Transpl..

[29] Orvain C., Wilson J.A., Fang M., Sandmaier B.M., Rodríguez-Arbolí E., Wood B.L., Othus M., Appelbaum F.R., Walter R.B. (2023). Relative impact of residual cytogenetic abnormalities and flow cytometric measurable residual disease on outcome after allogeneic hematopoietic cell transplantation in adult acute myeloid leukemia. Haematologica.

[30] Curran E., Stock W. (2019). Taking a “bite out of all”: Blinatumomab approval for MRD-positive ALL. Blood.

[31] Tettero J.M., Freeman S., Buecklein V., Venditti A., Maurillo L., Kern W., Walter R.B., Wood B.L., Roumier C., Philippé J. (2022). Technical Aspects of Flow Cytometry-based Measurable Residual Disease Quantification in Acute Myeloid Leukemia: Experience of the European LeukemiaNet MRD Working Party. Hemasphere.

[32] Ghannam J., Dillon L.W., Hourigan C.S. (2020). Next-generation sequencing for measurable residual disease detection in acute myeloid leukaemia. Br. J. Haematol..

[33] Vassault A. (2019). Interpretation and analysis of the requirements of the standard EN ISO 15189: 2012. Ann. Biol. Clin..

[34] Hanekamp D., Bachas C., van de Loosdrecht A., Ossenkoppele G., Cloos J. (2020). Re: Myeloblasts in normal bone marrows expressing leukaemia-associated immunophenotypes. Pathology.

[35] Röhnert M.A., Kramer M., Schadt J., Ensel P., Thiede C., Krause S.W., Bücklein V., Hoffmann J., Jaramillo S., Schlenk R.F. (2022). Reproducible measurable residual disease detection by multiparametric flow cytometry in acute myeloid leukemia. Leukemia.

[36] Wood B.L. (2020). Acute myeloid leukemia minimal residual disease detection: The difference from normal approach. Curr. Protoc. Cytom..

[37] Guénot C., Lacombe F., Allou K., Dumezy F., Feuillard J., Geneviève F., Guérin E., Guy J., Maynadié M., Wagner-Ballon O. (2019). Peripheral blood minimal/measurable residual disease assessed in flow cytometry in acute myeloblastic leukemia. Leukemia.

[38] Godwin C.D., Zhou Y., Othus M., Asmuth M.M., Shaw C.M., Gardner K.M., Wood B.L., Walter R.B., Estey E.H. (2021). Acute myeloid leukemia measurable residual disease detection by flow cytometry in peripheral blood vs bone marrow. Blood.

[39] Lacombe F., Bernal E., Bloxham D., Couzens S., Porta M.G.D., Johansson U., Kern W., Macey M., Matthes T., Morilla R. (2016). Harmonemia: A universal strategy for flow cytometry immunophenotyping—A European LeukemiaNet WP10 study. Leukemia.

[40] Zeijlemaker W., Grob T., Meijer R., Hanekamp D., Kelder A., Carbaat-Ham J.C., Oussoren-Brockhoff Y.J.M., Snel A.N., Veldhuizen D., Scholten W.J. (2019). CD34+CD38− leukemic stem cell frequency to predict outcome in acute myeloid leukemia. Leukemia.

[41] van Rhenen A., Moshaver B., Kelder A., Feller N., Nieuwint A.W.M., Zweegman S., Ossenkoppele G.J., Schuurhuis G.J. (2007). Aberrant marker expression patterns on the CD34+CD38- stem cell compartment in acute myeloid leukemia allows to distinguish the malignant from the normal stem cell compartment both at diagnosis and in remission. Leukemia.

[42] Li S.-Q., Xu L.-P., Wang Y., Zhang X.-H., Chen H., Chen Y.-H., Wang F.-R., Han W., Sun Y.-Q., Yan C.-H. (2022). An LSC-based MRD assay to complement the traditional MFC method for prediction of AML relapse: A prospective study. Blood.

[43] Rodríguez-Arbolí E., Othus M., Freeman S., Buccisano F., Ngai L.L., Thomas I., Palmieri R., Cloos J., Johnson S., Meddi E. (2024). Optimal prognostic threshold for acute myeloid leukemia (AML) measurable residual disease (MRD) positivity by multiparameter flow cytometry: A report of 2051 patients from MRC/NCRI, Gimema, HOVON, and Seattle. Transplant. Cell. Ther..

[44] Short N.J., Dillon R. (2025). Measurable residual disease monitoring in AML: Prospects for therapeutic decision-making and new drug development. Am. J. Hematol..

[45] Tobal K., Yin J.A.L. (1998). Molecular monitoring of minimal residual disease in acute myeloblastic leukemia with t(8;21) by RT-PCR. Leuk. Lymphoma.

[46] Freeman S.D., Hourigan C.S. (2019). MRD evaluation of AML in clinical practice: Are we there yet?. Hematology.

[47] Ivey A., Hills R.K., Simpson M.A., Jovanovic J.V., Gilkes A., Grech A., Patel Y., Bhudia N., Farah H., Mason J. (2016). Assessment of Minimal Residual Disease in Standard-Risk AML. N. Engl. J. Med..

[48] Rücker F.G., Agrawal M., Corbacioglu A., Weber D., Kapp-Schwoerer S., Gaidzik V.I., Jahn N., Schroeder T., Wattad M., Lübbert M. (2019). Measurable residual disease monitoring in acute myeloid leukemia with t(8;21)(q22;q22.1): Results from the AML Study Group. Blood.

[49] Boddu P., Gurguis C., Sanford D., Cortes J., Akosile M., Ravandi F., Garcia-Manero G., Patel K.P., Kadia T., Brandt M. (2018). Response kinetics and factors predicting survival in core-binding factor leukemia. Leukemia.

[50] Gabert J., Beillard E., Van der Velden V.H., Bi W., Grimwade D., Pallisgaard N., Barbany G., Cazzaniga G., Cayuela J.M., Cavé H. (2003). Standardization and quality control studies of ‘real-time’ quantitative reverse transcriptase polymerase chain reaction of fusion gene transcripts for residual. Leukemia.

[51] Hubmann M., Köhnke T., Hoster E., Schneider S., Dufour A., Zellmeier E., Fiegl M., Braess J., Bohlander S.K., Subklewe M. (2014). Molecular response assessment by quantitative real-time polymerase chain reaction after induction therapy in NPM1-mutated patients identifies those at high risk of relapse. Haematologica.

[52] Jain P., Kantarjian H., Patel K., Faderl S., Garcia-Manero G., Benjamini O., Borthakur G., Pemmaraju N., Kadia T., Daver N. (2014). Mutated NPM1 in patients with acute myeloid leukemia in remission and relapse. Leuk. Lymphoma.

[53] van der Velden V.H.J., Hochhaus A., Cazzaniga G., Szczepanski T., Gabert J., Van Dongen J.J.M. (2003). Detection of minimal residual disease in hematologic malignancies by real-time quantitative PCR: Principles, approaches, and laboratory aspects. Leukemia.

[54] Scott S., Dillon R., Thiede C., Sadiq S., Cartwright A., Clouston H.J., Travis D., Mokretar K., Potter N., Chantry A. (2023). Assessment of acute myeloid leukemia molecular measurable residual disease testing in an interlaboratory study. Blood Adv..

[55] Levis M.J., Hamadani M., Logan B., Jones R.J., Singh A.K., Litzow M., Wingard J.R., Papadopoulos E.B., Perl A.E., Soiffer R.J. (2024). Gilteritinib as Post-Transplant Maintenance for AML With Internal Tandem Duplication Mutation of FLT3. J. Clin. Oncol..

[56] Madaci L., Farnault L., Abbou N., Gabert J., Venton G., Costello R. (2023). Impact of Next-Generation Sequencing in Diagnosis, Prognosis and Therapeutic Management of Acute Myeloid Leukemia/Myelodysplastic Neoplasms. Cancers.

[57] Luo P., Jing W., Yi K., Wu S., Zhou F. (2020). Wilms’ tumor 1 gene in hematopoietic malignancies: Clinical implications and future directions. Leuk. Lymphoma.

[58] Lesieur A., Thomas X., Nibourel O., Boissel N., Fenwarth L., De Botton S., Fournier E., Celli-Lebras K., Raffoux E., Recher C. (2021). Minimal residual disease monitoring in acute myeloid leukemia with non-A/B/D NPM1 mutations by digital polymerase chain reaction: Feasibility and clinical use. Haematologica.

[59] Bacher U., Dicker F., Haferlach C., Alpermann T., Rose D., Kern W., Haferlach T., Schnittger S. (2014). Quantification of rare NPM1 mutation subtypes by digital PCR. Br. J. Haematol..

[60] Rausch C., Rothenberg-Thurley M., Buerger S.A., Tschuri S., Dufour A., Neusser M., Schneider S., Spiekermann K., Metzeler K.H., Ziemann F. (2021). Double Drop-Off Droplet Digital PCR: A Novel, Versatile Tool for Mutation Screening and Residual Disease Monitoring in Acute Myeloid Leukemia Using Cellular or Cell-Free DNA. J. Mol. Diagn..

[61] Blachly J.S., Walter R.B., Hourigan C.S. (2022). The present and future of measurable residual disease testing in acute myeloid leukemia. Haematologica.

[62] Zhao Z., Lan J. (2024). Detection methods and prognosis implications of measurable residual disease in acute myeloid leukemia. Ann. Hematol..

[63] Dillon L.W., Gui G., Page K.M., Ravindra N., Wong Z.C., Andrew G., Mukherjee D., Zeger S.L., El Chaer F., Spellman S. (2023). DNA sequencing to detect residual disease in adults with acute myeloid leukemia prior to hematopoietic cell transplant. JAMA.

[64] Yoest J.M., Shirai C.L., Duncavage E.J. (2020). Sequencing-based measurable residual disease testing in acute myeloid leukemia. Front. Cell Dev. Biol..

[65] Salk J.J., Schmitt M.W., Loeb L.A. (2018). Enhancing the accuracy of next-generation sequencing for detecting rare and subclonal mutations. Nat. Rev. Genet..

[66] Gaksch L., Kashofer K., Heitzer E., Quehenberger F., Daga S., Hofer S., Halbwedl I., Graf R., Krisper N., Hoefler G. (2018). Residual disease detection using targeted parallel sequencing predicts relapse in cytogenetically normal acute myeloid leukemia. Am. J. Hematol..

[67] Lee J.-M., Park S., Hwang I., Kang D., Cho B.S., Kim H.-J., Ahn A., Kim M., Kim Y. (2022). FLT3-ITD Measurable Residual Disease Monitoring in Acute Myeloid Leukemia Using Next-Generation Sequencing. Cancers.

[68] Heitzer E., Haque I.S., Roberts C.E.S., Speicher M.R. (2019). Current and future perspectives of liquid biopsies in genomics-driven oncology. Nat. Rev. Genet..

[69] Colmenares R., Álvarez N., Barrio S., Martínez-López J., Ayala R. (2022). The Minimal Residual Disease Using Liquid Biopsies in Hematological Malignancies. Cancers.

[70] Chen M., Zhao H. (2019). Next-generation sequencing in liquid biopsy: Cancer screening and early detection. Hum. Genom..

[71] Allam S., Nasr K., Khalid F., Shah Z., Suheb M.Z.K., Mulla S., Vikash S., Bou Zerdan M., Anwer F., Chaulagain C.P. (2023). Liquid biopsies and minimal residual disease in myeloid malignancies. Front. Oncol..

[72] Rolfo C., Cardona A.F., Cristofanilli M., Paz-Ares L., Diaz Mochon J.J., Duran I., Raez L.E., Russo A., Lorente J.A., Malapelle U. (2020). Challenges and opportunities of cfDNA analysis implementation in clinical practice: Perspective of the International Society of Liquid Biopsy (ISLB). Crit. Rev. Oncol. Hematol..

[73] Valls L., Badve C., Avril S., Herrmann K., Faulhaber P., O’Donnell J., Avril N. (2016). FDG-PET imaging in hematological malignancies. Blood Rev..

[74] Han E.J., Lee B.H., Kim J.A., Park Y.H., Choi W.H. (2017). Early assessment of response to induction therapy in acute myeloid leukemia using 18F-FLT PET/CT. EJNMMI Res..

[75] Vanderhoek M., Juckett M.B., Perlman S.B., Nickles R.J., Jeraj R. (2011). Early assessment of treatment response in patients with AML using [(18)F]FLT PET imaging. Leuk. Res..

[76] Study Details|NCT02392429|FLT PET/CT in Measuring Response in Patients with Previously Untreated Acute Myeloid Leukemia|ClinicalTrials.gov. NCT02392429.

[77] Pratz K.W., Jonas B.A., Pullarkat V., Recher C., Schuh A.C., Thirman M.J., Garcia J.S., DiNardo C.D., Vorobyev V., Fracchiolla N.S. (2022). Measurable residual disease response and prognosis in treatment-naïve acute myeloid leukemia with venetoclax and azacitidine. J. Clin. Oncol..

[78] Othman J., Tiong I.S., O’Nions J., Dennis M., Mokretar K., Ivey A., Austin M., Latif A.-L., Amer M., Chan W.Y. (2024). Molecular MRD is strongly prognostic in patients with NPM1-mutated AML receiving venetoclax-based nonintensive therapy. Blood.

[79] Bernardi M., Ferrara F., Carrabba M.G., Mastaglio S., Lorentino F., Vago L., Ciceri F. (2022). MRD in Venetoclax-Based Treatment for AML: Does it Really Matter?. Front. Oncol..

[80] Tiong I.S., Dillon R., Ivey A., Teh T.-C., Nguyen P., Cummings N., Taussig D.C., Latif A.-L., Potter N.E., Runglall M. (2021). Venetoclax induces rapid elimination of NPM1 mutant measurable residual disease in combination with low-intensity chemotherapy in acute myeloid leukaemia. Br. J. Haematol..

[81] Maiti A., Dinardo C.D., Wang S.A., Jorgensen J., Kadia T.M., Daver N.G., Short N.J., Yilmaz M., Pemmaraju N., Borthakur G. (2021). Prognostic value of measurable residual disease after venetoclax and decitabine in acute myeloid leukemia. Blood Adv..

[82] Ong S.Y., Tan Si Yun M., Abdul Halim N.A., Christopher D., Jen W.Y., Gallardo C., Tan Hwee Yim A., Woon Y.K., Ng H.J., Ooi M. (2022). Real-World Experience of Measurable Residual Disease Response and Prognosis in Acute Myeloid Leukemia Treated with Venetoclax and Azacitidine. Cancers.

[83] Ragon B.K., Daver N., Garcia-Manero G., Ravandi F., Cortes J., Kadia T., Oran B., Ohanian M., Ferrajoli A., Pemmaraju N. (2017). Minimal residual disease eradication with epigenetic therapy in core binding factor acute myeloid leukemia. Am. J. Hematol..

[84] Ravandi F., Cloos J., Buccisano F., Dillon R., Döhner K., Freeman S.D., Hourigan C.S., Ossenkoppele G.J., Roboz G.J., Subklewe M. (2023). Measurable residual disease monitoring in patients with acute myeloid leukemia treated with lower-intensity therapy: Roadmap from an ELN-DAVID expert panel. Am. J. Hematol..

[85] WaWalter R.B., Gooley T.A., Wood B.L., Milano F., Fang M., Sorror M.L., Estey E.H., Salter A.I., Lansverk E., Chien J.W. (2011). Impact of pretransplantation minimal residual disease, as detected by multiparametric flow cytometry, on outcome of myeloablative hematopoietic cell transplantation for acute myeloid leukemia. J. Clin. Oncol..

[86] Araki D., Wood B.L., Othus M., Radich J.P., Halpern A.B., Zhou Y., Mielcarek M., Estey E.H., Appelbaum F.R., Walter R.B. (2016). Allogeneic hematopoietic cell transplantation for acute myeloid leukemia: Time to move toward a minimal residual disease-based definition of complete remission?. J. Clin. Oncol..

[87] Jentzsch M., Grimm J., Bill M., Brauer D., Backhaus D., Schulz J., Goldmann K., Niederwieser D., Platzbecker U., Schwind S. (2021). Prognostic relevance of remission and measurable residual disease status in AML patients prior to reduced intensity or non-myeloablative allogeneic stem cell transplantation. Blood Cancer J..

[88] Gilleece M.H., Shimoni A., Labopin M., Robinson S., Beelen D., Socié G., Unal A., Ganser A., Vitek A., Sengeloev H. (2021). Measurable residual disease status and outcome of transplant in acute myeloid leukemia in second complete remission: A study by the acute leukemia working party. Blood Cancer J..

[89] Jentzsch M., Bischof L., Backhaus D., Brauer D., Schulz J., Franke G.-N., Vucinic V., Niederwieser D., Platzbecker U., Schwind S. (2022). Impact of MRD status in patients with AML undergoing allogeneic stem cell transplantation in the first vs. the second remission. Blood Adv..

[90] Venditti A., Piciocchi A., Candoni A., Melillo L., Calafiore V., Cairoli R., de Fabritiis P., Storti G., Salutari P., Lanza F. (2019). GIMEMA AML1310 trial of risk-adapted, MRD-directed therapy for young adults with newly diagnosed acute myeloid leukemia. Blood.

[91] Balsat M., Renneville A., Thomas X., De Botton S., Caillot D., Marceau A., Lemasle E., Marolleau J.P., Nibourel O., Berthon C. (2017). Postinduction Minimal Residual Disease Predicts Outcome and Benefit from Allogeneic Stem Cell Transplantation in Acute Myeloid Leukemia with NPM1 Mutation. J. Clin. Oncol..

[92] Venditti A., Peter Gale R., Buccisano F., Ossenkoppele G. (2020). Should persons with acute myeloid leukemia (AML) in 1st histological complete remission who are measurable residual disease (MRD) test positive receive an allotransplant?. Leukemia.

[93] Paras G., Morsink L.M., Othus M., Milano F., Sandmaier B.M., Zarling L.C., Palmieri R., Schoch G., Davis C., Bleakley M. (2022). Conditioning intensity and peritransplant flow cytometric MRD dynamics in adult AML. Blood.

[94] Walter R.B., Gyurkocza B., Storer B.E., Godwin C.D., Pagel J.M., Buckley S.A., Sorror M.L., Wood B.L., Storb R., Appelbaum F.R. (2015). Comparison of minimal residual disease as outcome predictor for AML patients in first complete remission undergoing myeloablative or nonmyeloablative allogeneic hematopoietic cell transplantation. Leukemia.

[95] Hourigan C.S., Dillon L.W., Gui G., Logan B.R., Fei M., Ghannam J., Li Y., Licon A., Alyea E.P., Bashey A. (2020). Impact of Conditioning Intensity of Allogeneic Transplantation for Acute Myeloid Leukemia with Genomic Evidence of Residual Disease. J. Clin. Oncol..

[96] Chang Y.-J., Wang Y., Liu Y.-R., Xu L.-P., Zhang X.-H., Chen H., Chen Y.-H., Wang F.-R., Han W., Sun Y.-Q. (2017). Haploidentical allograft is superior to matched sibling donor allograft in eradicating pre-transplantation minimal residual disease of AML patients as determined by multiparameter flow cytometry: A retrospective and prospective analysis. J. Hematol. Oncol..

[97] Platzbecker U., Middeke J.M., Sockel K., Herbst R., Wolf D., Baldus C.D., Oelschlägel U., Mütherig A., Fransecky L., Noppeney R. (2018). Measurable residual disease-guided treatment with azacitidine to prevent haematological relapse in patients with myelodysplastic syndrome and acute myeloid leukaemia (RELAZA2): An open-label, multicentre, phase 2 trial. Lancet Oncol..

[98] Loke J., McCarthy N., Jackson A., Siddique S., Hodgkinson A., Mason J., Crawley C., Gilleece M., Peniket A., Protheroe R. (2023). Posttransplant MRD and T-cell chimerism status predict outcomes in patients who received allografts for AML/MDS. Blood Adv..

[99] Jackson J.M., Taylor J.B., Witek M.A., Hunsucker S.A., Waugh J.P., Fedoriw Y., Shea T.C., Soper S.A., Armistead P.M. (2016). Microfluidics for the detection of minimal residual disease in acute myeloid leukemia patients using circulating leukemic cells selected from blood. Analyst.

[100] Platzbecker U., Wermke M., Radke J., Oelschlaegel U., Seltmann F., Kiani A., Klut I.-M., Knoth H., Röllig C., Schetelig J. (2012). Azacitidine for treatment of imminent relapse in MDS or AML patients after allogeneic HSCT: Results of the RELAZA trial. Leukemia.

[101] Burchert A., Bug G., Fritz L.V., Finke J., Stelljes M., Röllig C., Wollmer E., Wäsch R., Bornhäuser M., Berg T. (2020). Sorafenib Maintenance After Allogeneic Hematopoietic Stem Cell Transplantation for Acute Myeloid Leukemia with FLT3-Internal Tandem Duplication Mutation (SORMAIN). J. Clin. Oncol..

[102] Cortes J.E., Khaled S., Martinelli G., Perl A.E., Ganguly S., Russell N., Krämer A., Dombret H., Hogge D., Jonas B.A. (2019). Quizartinib versus salvage chemotherapy in relapsed or refractory FLT3-ITD acute myeloid leukaemia (QuANTUM-R): A multicentre, randomised, controlled, open-label, phase 3 trial. Lancet Oncol..

[103] Dholaria B., Savani B.N., Labopin M., Luznik L., Ruggeri A., Mielke S., Al Malki M.M., Kongtim P., Fuchs E., Huang X.-J. (2020). Clinical applications of donor lymphocyte infusion from an HLA-haploidentical donor: Consensus recommendations from the Acute Leukemia Working Party of the EBMT. Haematologica.

